# Functional connectivity differences in patients with mood disorders: an exploratory fMRI study comparing electroconvulsive therapy with pharmacological treatment

**DOI:** 10.1016/j.nsa.2025.105522

**Published:** 2025-06-11

**Authors:** Lydia Fortea, Alexander T. Ysbæk-Nielsen, Jeff Zarp, Patrick M. Fisher, Lars Kessing, Martin Balslev Jørgensen, Gitte Moos Knudsen, Joaquim Radua, Eduard Vieta, Julian Macoveanu, Kamilla W. Miskowiak

**Affiliations:** a Institut d’Investigacions Biomèdiques August Pi I Sunyer (IDIBAPS), C/ del Rosselló, 149, L'Eixample, 08036, Barcelona, Spain; b Department of Medicine, Institute of Neuroscience, University of Barcelona, Gran Via de les Corts Catalanes, 585, L'Eixample, 08007, Barcelona, Spain; c Neurobiology Research Unit, Rigshospitalet, Blegdamsvej 9, København, 2100, Copenhagen, Denmark; d Copenhagen Affective disorder Research Center (CADIC), Psychiatric Centre Copenhagen, Mental Health Services, Capital Region of Denmark, Nordre Fasanvej 59, Frederiksberg, 2000, Copenhagen, Denmark; e Neurocognition and Emotion in Affective Disorders Centre (NEAD), Department of Psychology, University of Copenhagen, and Psychiatric Centre Copenhagen, Mental Health Services, Capital Region of Denmark, Nørregade 10, København, 1172, Copenhagen, Denmark; f Department of Drug Design and Pharmacology, University of Copenhagen, Nørregade 10, København, 1172, Copenhagen, Denmark; g Department of Clinical Medicine, University of Copenhagen, Denmark; h Centro de Investigación Biomédica en Red de Salud Mental (CIBERSAM), Instituto de Salud Carlos III, Av. de Monforte de Lemos, 5, Fuencarral-El Pardo, 28029, Madrid, Spain; i Bipolar and Depressive Disorders Unit, Hospital Clinic, C. de Villarroel, 170, L'Eixample, 08036, Barcelona, Spain

**Keywords:** Electroconvulsive therapy, Mood disorder, Functional connectivity, Cognition, Depressive symptoms

## Abstract

Electroconvulsive therapy (ECT) has been shown to induce widespread dysregulation of network connectivity in patients with mood disorders. Nevertheless, the extent to which these functional changes contribute to patients’ cognitive side-effects or depressive symptoms improvement remains unclear. This study investigated cross-sectional resting-state functional connectivity (rs-FC) differences in patients with mood disorders after their 8th ECT session (ECT group, n = 33) compared to those receiving pharmacological treatment (non-ECT group, n = 36) and healthy controls (n = 34). Furthermore, we explored the association of rs-FC differences with cognitive side-effects and depressive symptom improvements assessed longitudinally in the ECT group. We focused on analyzing rs-FC within- and between the default mode network (DMN), executive control network (ECN), and frontoparietal network (FPN). Additionally, we explored the association between significant rs-FC group differences and verbal memory decline, and depressive symptoms improvement from pre-to post-ECT within the ECT group. ECT-treated patients exhibited *hyper-*connectivity within the left-hemisphere FPN compared to those on pharmacological treatment, along with *hypo-*connectivity between ECN and FPN (p-corrected<0.02). Depressive symptoms positively correlated with rs-FC within the right-hemisphere FPN (p-corrected<0.04). Notably, rs-FC differences were unrelated to verbal memory decline or symptom improvement from pre-to post-ECT (p-corrected>0.1). Our findings highlight differences in brain connectivity between remitted patients after ECT and diagnosis-matched patients following standard pharmacological treatment. Further studies are warranted to investigate longitudinal rs-FC effects of ECT to identify biomarkers predictive of treatment response and the risk of cognitive side effects after ECT.

## Highlights

• ECT-treated patients showed higher RS-FC within the FPN-left compared to non-ECT.
• ECT-treated patients showed lower RS-FC between ECN and FPN compared to non-ECT.
• RS-FC differences did not correlate with cognitive decline after ECT.

## 1 Introduction

Electroconvulsive therapy (ECT) is a highly effective treatment for treatment-resistant or severely depressed individuals with bipolar disorder (BD) or major depressive disorder (MDD) (UK ECT Review Group, 2003), achieving a fast symptom remission in approximately 50–70 % of patients (Kellner et al., 2012; Husain et al., 2004). However, the use of ECT remains controversial, largely due to its common cognitive side-effects on memory, attention, and executive functions, generally confined to the first three days following treatment completion (Semkovska and McLoughlin, 2010). Yet, findings of the pattern, severity, and persistence of these cognitive side effects are inconsistent (Mervaala et al., 2001; Schat et al., 2007; Fujita et al., 2006; Perera et al., 2004). In addition, ECT is a stigmatized intervention, partly due to negative cultural depictions in the media (Sienaert, 2016) and the limited understanding of its underlying neurobiological mechanisms. This limitation in knowledge is partially due to methodological challenges in assessing cognition in severely depressed patients with mood disorders. The use of comprehensive neurocognitive test batteries in this population often leads to high attrition and significant drop-out rates, further complicating research efforts (Landry et al., 2021). This bias, which may lead to underestimation of ECT-related cognitive side effects, can be mitigated by administering short neurocognitive screening instruments, such as the Screen for Cognitive Impairment in Psychiatry (SCIP) (Hammershøj et al., 2022). However, this approach with such relatively crude instruments comes at the expense of a detailed insight into the pattern of side effects.

Emerging evidence from resting-state functional magnetic resonance imaging (rs-fMRI) studies suggests that ECT improves patients’ mood state by modulating the resting-state functional connectivity (rs-FC) of brain networks, including the default mode network (DMN), executive central network (ECN), and frontoparietal network (FPN) (Wei et al., 2018; Mulders et al., 2016; Porta-Casteràs et al., 2021; Belge et al., 2021). Notably, abnormal communication within and between the internal attention network, like the DMN, and the goal-directed networks, such as the ECN and FPN, has been frequently reported as core features of depression (Brandl et al., 2022; Kaiser et al., 2015; Sha et al., 2019). Moreover, increased rs-FC within the DMN may also be a neuronal substrate of the illness-associated cognitive impairments commonly seen in mood disorders (Fortea et al., 2023). However, it is worth noting that while a hyper-connected DMN is consistently linked to depression (Berman et al., 2011; Connolly et al., 2013), treatment-resistant depression appears to be associated with hypo-connectivity within the DMN (Runia et al., 2022).

Regarding insight into the neurobiological effects of ECT, numerous studies have highlighted widespread rs-FC changes in patients with severe depression following ECT treatment (Wei et al., 2018; Mulders et al., 2016; Porta-Casteràs et al., 2021; Belge et al., 2021; Perrin et al., 2012). Some have reported increased rs-FC within the DMN after ECT (Wei et al., 2018; Porta-Casteràs et al., 2021; Belge et al., 2021), albeit this was associated with neither mood improvement nor cognitive decline (Wei et al., 2018). Additionally, reductions in global rs-FC in the dorsolateral prefrontal cortex (dlPFC), a key region of the ECN, have been observed post-ECT, correlating with patients' symptom improvement (Perrin et al., 2012). Other studies have reported normalization of rs-FC between the DMN and ECN, which may reflect a restoration of network dynamics disrupted in depression (Belge et al., 2021; Wang et al., 2018; Yrondi et al., 2018). Notably, some studies found that ECT led to bidirectional structural and rs-FC changes between the amygdala and other cortical and subcortical structures in patients with MDD or schizophrenia, suggesting that ECT-related functional changes may not be diagnosis-specific but rather reflect broader neuromodulatory effects on the brain (Wolf et al., 2016; Thomann et al., 2017). Further, two studies have linked ECT-induced rs-FC changes among FPN, DMN, and subcortical structures, such as the hippocampus, to cognitive impairment following ECT, suggesting that verbal memory decline may be associated with disrupted communication among those networks (Wei et al., 2018; Wang et al., 2020; Qi et al., 2022)**.** Despite these insights, there is a limited understanding of the neural mechanism underlying ECT-related cognitive side effects. Further research is necessary to delve deeper into the rs-FC alterations associated with impaired cognitive function post-ECT and their relationship with depressive symptoms.

This explorative rs-fMRI study aimed to delineate the rs-FC differences associated with ECT treatment and depressive symptoms by (I) comparing rs-FC between depressed patients with mood disorders post-ECT and age, sex and diagnosis-matched pharmacological-treated out-patients with mood disorders in full or partial remission; (II) exploring the associations between depressive symptoms and rs-FC across both patient groups; and (III) assess whether any identified rs-FC differences are associated with cognitive decline and improvement of depressive symptom within the ECT group.

## 2 Material and methods

### 2.1 Study design and participants

This study pooled rs-fMRI, clinical and neurocognitive data from three study cohorts (Schmidt et al., 2018; Kessing et al., 2017; Petersen et al., 2018; Ott et al., 2018), previously used by Macoveanu et al. (2024): 36 severely depressed patients with MDD or BD who received ECT, 37 pharmacological-treated patients with MDD or BD in full or partial remission, and 34 healthy controls (HC). Groups were matched for sex and age. One ECT and three non-ECT patients were excluded due to high head movement. All participants were recruited at the Psychiatric Centre Copenhagen, using the same MRI scanner at the Copenhagen University Hospital, Rigshospitalet, acquisition methods, and neurocognitive and depressive symptoms assessments. The Danish Research Ethics Committee approved original studies for the Capital Region of Denmark (EPO-T: H-16038506; PRETEC: H-16043370; BIO: H-7–2014–007).

The ECT group was pooled from the EPO-T study (NCT0333959627 (Schmidt et al., 2018),). It consisted of in-patients aged 18–70 years with MDD or BD according to ICD-10 criteria (verified with the Mini International Neuropsychiatric Interview (MINI) (Lecrubier et al., 1997)), with moderate to severe depressive episode symptoms (Hamilton Depression Rating Scale 17-items (HDRS-17) >17 (Hamilton, 1960)). All patients fulfilled the criteria for ECT intervention, treatment-resistant depression, and/or sufficiently severe depression (Posternak et al., 2004; Sackeim, 2001). Patients with severe psychotic depression, who were unable to provide written consent, or with acute suicidal risk were excluded. Participants underwent a total of eight sessions of bilateral ECT (post-ECT), as opposed to the typical 12 (Porter et al., 2020), to minimize attrition rates and prevent floor effects of cognitive measures (e.g., a slowdown in cognitive decline overtreatment). Indeed, previous neuroimaging studies on ECT have shown that eight sessions are sufficient to induce observable changes in the brain (Gryglewski et al., 2019). This approach enables us to thoroughly examine the cognitive side effects and mood improvement following ECT. Participants completed a neuropsychological assessment before and three days post-ECT and a single MRI scan two to five days post-ECT. The original clinical trial investigated the effects of add-on erythropoietin (EPO) versus saline treatment on cognitive side effects of ECT (Schmidt et al., 2018). They found significant group differences in pre-defined cognitive outcomes or neural activity patterns (data not shown) (Miskowiak et al., 2024). Thus, we decided to combine data from both groups.

The non-ECT group was pooled from the BIO (NCT02888262 (Kessing et al., 2017)) and PRETEC studies (NCT03315897 (Petersen et al., 2018); NCT03814122 (Ott et al., 2018)). It included out-patients aged 18–65 years with BD or MDD according to ICD-10 criteria (verified with the Schedules for Clinical Assessment in Neuropsychiatry (SCAN) (Gryglewski et al., 2019), in full or partial remission at the time of assessment (HDRS-17 and Young Mania Rating Scale (YMRS) ≤7 or ≤14, respectively (Hamilton, 1960; Young et al., 1978)), and receiving standard pharmacological psychotropic treatment. Non-ECT patients were assessed cross-sectionally with neuropsychological tests and rs-fMRI before their enrollment in the observational study (Kessing et al., 2017) or as part of a cognitive intervention study. Healthy controls, aged 18 to 65, were recruited from the BIO study (Kessing et al., 2017). They were only assessed cross-sectionally with neuropsychological tests and rs-fMRI. They had no personal or first-degree relative history of treatment-required psychiatric disorder or neurological illness.

Exclusion criteria for all participants included a history of severe brain injury, current alcohol or substance use disorder (within the past three months), diagnosis of dyslexia, pregnancy, or severe somatic illness.

### 2.2 Cognitive assessment

Cognition was assessed with a comprehensive neurocognitive test battery that included Trail Making Test Part B (TMT-B) (Office AG, 1944), Rey Auditory Verbal Learning Test (RAVLT) (Schmidt, 1996), Repeatable Battery for the Assessment of Neuropsychological Status (RBANS) Coding (Randolph et al., 1998), Verbal Fluency (letter ‘D’) (Borkowski et al., 1967), Letter-Number Sequencing subtest from the Wechsler Adult Intelligence Scale (WAIS-III) (Wechsler, 1997), and the Rapid Visual Information Processing (RVP) from CANTAB (Cambridge Cognition Ltd.) (Sahakian and Owen, 1992). Verbal intelligence was estimated in the non-ECT patient group using the Danish Adult Reading Task (DART) (Nelson and O'Connell, 1978).

For the non-ECT and ECT groups (pre- and post-ECT, separately), we transformed raw scores from the neuropsychological test measures to *z*-scores based on the mean and standard deviation from the HC group. These *z*-scores were then averaged and grouped into four cognitive domains: ‘processing speed’, ‘sustained attention,’ verbal learning and memory, and ‘working memory.’ Additionally, a global cognitive composite score was calculated by averaging the *z*-scores of these four cognitive domains.

### 2.3 Statistical analysis of behavioral data

Statistical analyses of behavioral data were performed using Statistical Package for the Social Sciences (SPSS), version 25 (IBM Corporation, Armonk, NY). Differences in demographic, clinical, and neurocognitive scores among the ECT and non-ECT patient groups and HC were assessed using analysis of variance (ANOVA) followed by Fisher's Least Significant Difference (LSD) post-hoc tests. Additionally, we analyzed the ECT group's pre- and post-treatment cognitive and clinical symptoms data to evaluate changes following ECT.

### 2.4 rs-fMRI acquisition and pre-processing

MRI data were collected using a 3 T S Prisma scanner (Siemens Trio, Erlangen, Germany) with a 64-channel head-neck coil. Detailed imaging parameters are provided in Supplementary Material.

The rs-fMRI data was pre-processed using FSL (www.fsl.fmrib.ox.ac.uk/fsl) (Jenkinson et al., 2012), including motion correction, high-pass temporal filtering (cut-off frequency 0.1 Hz), removal of non-brain tissue, co-registration to individual structural image, normalization to MNI (Montreal Neurologic Institute) standard space with 2 mm isotropic voxel size, and spatial smoothing with using a 5 mm Gaussian kernel. Functional data were denoised using the FIX classifier (Griffanti et al., 2014; Salimi-Khorshidi et al., 2014), which separates noise from resting-state networks (RSN) activity. The classifier, previously trained on a cohort with the same acquisition protocol (Fortea et al., 2023), was refined using data from seven participants in the current cohort. Participants with excessive head motion were excluded from further analysis (average relative framewise movement >0.25 mm).

### 2.5 rs-fMRI statistical analysis

Group-level independent component analysis (ICA) was conducted using Multivariate Exploratory Linear Optimized Decomposition into Independent Components (MELODIC), with the dimensionality set to 20 components (Beckmann et al., 2005; Smith et al., 2009). Identified components were compared with publicly available RSNs from the BrainMap database (Fox and Lancaster, 2002) through spatial correlation (FSL's *fslcc*), focusing on the pre-selected networks: DMN, ECN, and FPN (Smith et al., 2009). Due to its strong lateralization, FPN was analyzed as separate right and left hemisphere networks. The components showing the strongest correlation were selected for further analysis.

Then, we used a multivariate regression (*dual regression*) to obtain a subject-specific time series and spatial map [parameter estimate (PE) images] for each RSN (Beckmann et al., 2009). Within-RSN analysis included (i) calculation of RSN integrity by averaging the PE values across voxels within the RSN, followed by pairwise t-tests between patient groups; and (ii) voxel-wise comparison for each RSN with significant group differences, using non-parametric permutation testing (*randomise)* with 5000 permutations and Threshold-free cluster enhancement (TFCE) correction (Winkler et al., 2014; Smith et al., 2013). Between-RSN analyses utilized FSLNETs (https://fsl.fmrib.ox.ac.uk/fsl/fslwiki/FSLNets to calculate pairwise correlations between RSN's time series, followed by pairwise t-tests between patient groups. Regression analyses were used to compute the association between rs-FC and depressive symptoms. Statistical analyses were conducted with and without adjusting for depressive symptoms, and significance was set at family-wise error (FWE) corrected p < 0.05. Cluster significance for the voxel-wise analyses was set to k > 20 voxels to minimize the noise or artifacts at cluster-corrected-p < 0.05 divided by the number of studied RSN (e.g., for four RSN, p < 0.05/4 < 0.0125). This approach is consistent with methodologies commonly used in other rs-FC studies investigating ECT (Mulders et al., 2016; Belge et al., 2021). As a sensitivity analysis, we repeated the main group comparison, incorporating a covariable to account for medication use (yes/no), including anticonvulsants, antidepressants, antipsychotics, benzodiazepines, and lithium. Finally, we also compared treatment responders within the ECT group to non-ECT patients.

### 2.6 Correlation analyses

Pearson correlations were computed exclusively within the ECT group to examine associations between cognitive decline or improvements in depressive symptoms and significant rs-FC differences observed between patient groups, accounting for depressive symptoms as a covariate. Within-RSN, rs-FC variables included RSN integrity values and mean rs-FC strength from significant clusters. All statistical analyses were performed using SPSS, with significance determined by Bonferroni correction at *p* < 0.05.

## 3 Results

### 3.1 Participants characteristics

The final dataset comprised 69 patients (33 ECT and 36 non-ECT) and 34 HC. Demographic and clinical data comparisons among HC, non-ECT, and post-ECT groups are summarized in Table 1. Both patient groups were matched for mood disorder diagnoses (*p* = 0.3). Despite undergoing eight ECT sessions, ECT patients exhibited more severe depressive symptoms than non-ECT patients (HDRS-17, ECT: 17.8 ± 9, non-ECT: 7.6 ± 4.7; *p* < 0.001). Further, a higher proportion of ECT patients were prescribed more psychotropic medications during treatment (*p* = 0.006), specifically antipsychotics (52 % vs. 8 %, *p*_*FWE*_<0.001).

**Table 1:** Group differences in demographic, clinical, and cognitive characteristics of patients with mood disorders after 8 sessions of ECT, those who underwent standard pharmacological treatment, and healthy controls.

	ECT (n = 33)	Non-ECT (n = 36)	HC (n = 34)	F or X2	*p*	HC vs ECT	HC vs non-ECT (*p*)	ECT vs non-ECT (*p*)
**Demographics**								

Age *mean (SD)*	37.45 (13.3)	35.50 (11.1)	34.88 (10.4)	0.45	0.64	–	–	–
Sex *n females (%)*	25 (76)	26 (78)	24 (71)	0.24	0.89	–	–	–
Years of education. *mean (SD)*	15.48 (3.3)	14.78 (2.5)	16.45 (2.1)	3.74	0.028	**0.0035**	0.219	0.394

**Cognitive domain**								

Processing speed	−1.34	−0.88	0	13.28	**0.002**	**<0.001**	**<0.001**	0.097
Sustained attention	−0.39	−0.51	0	2.67	0.029	–	–	–
Verbal learning	−1.16	−0.64	0	8.34	**<0.001**	**<0.001**	0.012	0.101
Working memory	−1.16	−0.52	0	17.59	**<0.001**	**<0.001**	0.005	**0.003**
Global cognition	−1.04	−0.64	0	14.57	**<0.001**	**<0.001**	**<0.001**	0.061

**Clinical characteristics**								

HDRS-17 mean (SD)^a^	17.81 (9.3)	7.56 (4.7)	–					**<0.001**
Illness duration *mean years (SD)*	15.09 (10.6)	16.63 (11.3)	–					0.601
MDD *n (%)*	25 (76)	23 (64)						
BD *n (%)*	8 (24)	13 (36)	–					0.309
BD type II *n (%)*	3 (37)	10 (77)						0.179

**Medication status *n (%)***								

Antidepressants	25 (76)	15 (42)	–					0.015
Antipsychotics	17 (52)	3 (8)	–					**<0.001**
Anticonvulsants	10 (30)	10 (28)	–					0.999
Lithium	9 (27)	8 (22)	–					0.781
**Benzodiazepines**^b^^,^^c^	**10 (30)**	**3 (13)**						**0.200**

**Total number of medication classes *n (%)***								**0.006**

0	2 (6)	15 (42)	–					
1	11 (33)	9 (25)	–					
2	13 (39)	10 (28)	–					
3	5 (15)	1 (3)	–					
4	2 (6)	1 (3)	–					

Data in bold: Bonferroni corrected, α ≤ 0.05.

Footnote: ECT: electroconvulsive therapy; HDRS-17: Hamilton Depression Rating Scale 17-items; SD: standard deviation.

a Data missing for 2 subjects from the ECT group.

b Data missing for 13 subjects from the non-ECT group.

c Benzodiazepines were taken as needed but not on a daily basis.

Regarding cognitive performance, both patient groups showed significantly worse scores in ‘processing speed’ and global cognition than HC (*p*_*FWE*_<0.001). ECT patients also demonstrated poorer ‘working memory’ performance compared to both non-ECT patients and HC (p_FWE_<0.003) and exhibited lower’ verbal learning and memory’ scores relative to HC (p_FWE_<0.001).

Within the ECT group (see Table S1), depressive symptoms improved significantly from pre-to post-treatment (difference mean = 9.5, *p* < 0.001). Notably, 18 % of the patients achieved full clinical remission (HDRS ≤7), while another 18 % met the criteria for partial remission (HDRS ≤14). However, when comparing pre-and post-ECT cognitive scores, a significant decline was observed in ′verbal learning and memory’ (*p*_*FWE*_ = 0.03), with no significant changes in other cognitive domains or global cognition.

### 3.2 RS-FC analysis

#### 3.2.1 ICA components

We selected the ICA components with the highest correlation with our four networks of interest (Supplementary Fig. S1). The DMN was represented by the third component (*r* = 0.47), comprising the ventromedial PFC (vmPFC), precuneus, rostral anterior cingulate cortex (ACC), posterior cingulate cortex (PCC), and angular gyrus. The ECN was represented by the sixteenth component (*r* = 0.51) comprising the dlPFC, dorsal ACC, precuneus, insula, and thalamus. FPN-right and left were represented by the fifth and eighth components (*r* = 0.61 and 0.68, respectively), including the right and left PFC, medial PFC, superior occipital cortex, and middle temporal gyrus.

#### 3.2.2 Effect of ECT

The unadjusted analyses revealed a higher significant integrity value and voxel-wise hyper-connectivity within DMN, FPN-right, and FPN-left in ECT patients compared with non-ECT patients (*p*_*FWE*_<0.022, Table 2). In addition, they also showed higher rs-FC between DMN and FPN-right, along with lower rs-FC between ECN and FPN. Further details of unadjusted analysis are presented in the Supplement, Table S2, and Fig. S2.

**Table 2:** Integrity values for every studied network, DMN, ECN, FPN-left and FPN-right for patients following 8 sessions of ECT and those who underwent standard pharmacological treatment comparison. The group comparison was carried out without and with adjusting by depressive symptom severity and corrected for multiple comparison by Bonferroni (p_FWER_).

RSN	ECT	Non-ECT	No adjusted analysis		Adjusted analysis	
	Mean (SD)	Mean (SD)	t	p_FWER_	t	p_FWER_
DMN	14.71 (4.43)	11.69 (4.28)	2.87	0.022	−1.84	0.210
ECN	8.49 (2.86)	9.53 (4.33)	−1.19	0.950	1.11	0.864
FPN-left	13.23 (4.69)	9.84 (4.16)	3.14	0.011	−2.46	0.048
FPN-right	12.92 (4.58)	9.84 (3.92)	2.99	0.016	−1.79	0.234

Footnote: DMN: Default mode network; ECN: executive central network; ECT: electroconvulsive therapy; FPN: frontoparietal network; SD: standard deviation.

**Fig. 1:**
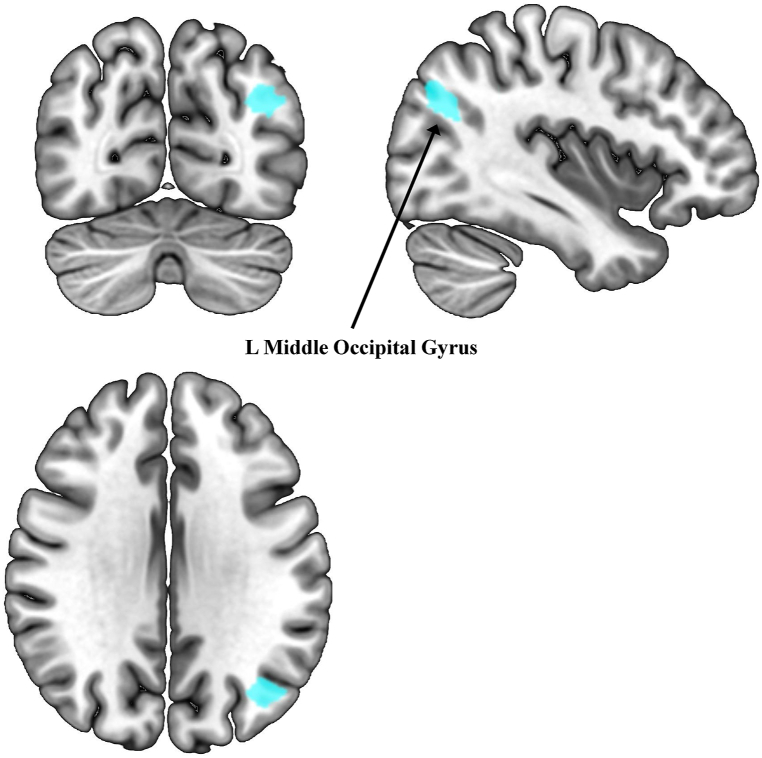
Brain regions from the frontoparietal network showing significantly higher within resting state functional connectivity in patients post-ECT compared to patients with traditional pharmacological treatment. Analysis was adjusted by depressive symptom severity and voxel corrected. Footnote: ECT: electroconvulsive therapy.

**Fig. 2:**
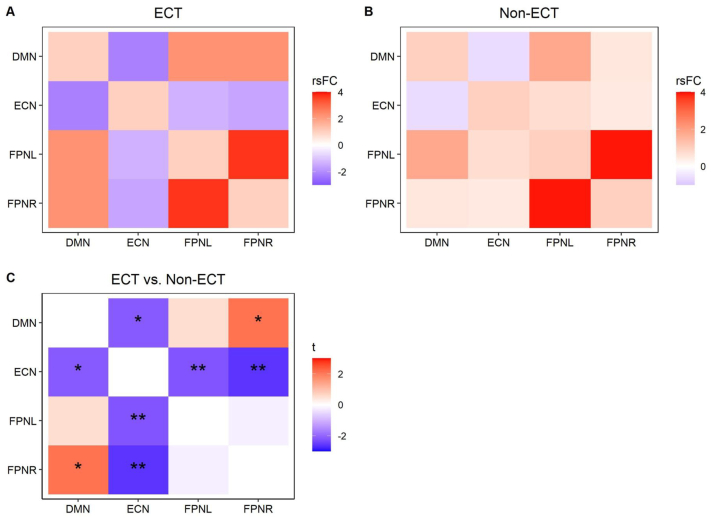
Heatmaps showing the resting-state functional connectivity between DMN, ECN, FPN-left, and FPN-right in patients following eight sessions of ECT (A), those who underwent standard pharmacological treatment (B), as well as the comparison between both groups (C). Analyses were adjusted by depressive symptom severity and corrected for multiple comparisons. Footnote: DMN: Default mode network; ECN: executive central network; ECT: electroconvulsive therapy; FPN: frontoparietal network. ∗ Significant at uncorrected value p < 0.05 ∗∗ Significant at FDR corrected value p < 0.05.

Analysis of network integrity after covarying for depressive symptoms only showed a significant group difference in the FPN-left after Bonferroni correction (*p*_*FWE*_ = 0.048, Table 2). In the voxel-wise analysis for FPN-left, ECT-treated patients showed greater positive rs-FC within the left middle occipital gyrus (x = −40, y = −70, z = −30, t = 4.32, p < 0.013) compared to non-ECT-treated patients (Fig. 1). This cluster remained significant when we adjusted for different medications (*p*_*FWE*_<0.034, Table S3). Further, ECT-treated patients showed *hypo-*connectivity between ECN and FPN-left and right (*p < 0.007*), between ECN and DMN (p = 0.03), and *hyper*-connectivity between DMN and FPN-right (p = 0.03) compared to non-ECT patients (Fig. 2). However, only the *hypo*-connectivity between ECN and FPN survived correction for multiple comparisons. In the sensitivity analysis accounting for medication use, significant group differences were only observed between ECN and FPN-right.

The comparison between individuals in the ECT-treated groups who responded to the treatment (n = 12) and those from the non-ECT group is presented in Table S4. No significant results were found in the within-RSN analysis and between-RSN after correction for multiple comparisons.

#### 3.2.3 Effect of depressive symptoms

A significant positive correlation was observed between depressive symptoms and network integrity in the DMN and FPN-right (*p*_*FWE*_<0.04). Voxel-wise analyses revealed a single cluster within the FPN-right, specifically in the right frontal medial orbital (x = 40, y = 50, z = −6, t = 3.71, *p* = 0.009), that presented a positive correlation with depressive symptoms. However, no significant association was found between depressive symptoms and between-RSN rs-FC.

#### 3.2.4 Association of rs-FC with cognitive decline and symptoms improvement in ECT patients

No significant association was found between the rs-FC differences observed between patient groups and the decline in ‘verbal learning and memory’ performance or the improvement of depression symptoms after treatment in ECT patients.

## 4 Discussion

This explorative resting-state fMRI study explored brain connectivity differences between post-ECT patients (n = 33) and pharmacologically treated patients (n = 36) and their association with cognitive side effects and depressive symptom improvement in the ECT group. Clinically, ECT patients showed a substantial alleviation of depressive symptoms after eight ECT sessions, although only 27 % achieved full or partial clinical remission. They also displayed a selective cognitive decline in verbal learning and memory. Neuroimaging revealed that ECT patients exhibited *hyper*-connectivity within the FPN-left and *hypo*-connectivity between ECN and FPN compared to non-ECT patients after adjusting for depressive symptoms. However, these rs-FC group differences were not associated with pre- to post-ECT verbal learning and memory decline or depressive symptom improvement. Additionally, residual depressive symptoms were positively associated with *hyper*-connectivity within the FPN-right. Therefore, although ECT patients displayed different rs-FC patterns compared to non-ECT patients, these differences appeared primarily related to depressive symptoms severity rather than cognitive side effects.

The rs-FC differences observed in the FPN-left in ECT-treated patients align with previous observations of increased connectivity in similar neural networks post-ECT (Belge et al., 2021; Wang et al., 2018). Such rs-FC differences may contribute to cognitive control deficits commonly observed in depressed states of mood disorders (Snyder, 2013) and deficits within memory and executive function (Mervaala et al., 2001; Fujita et al., 2006) because the FPN is crucial for such higher cognitive functions. Disruptions in the connectivity of this network may lead to difficulties in goal-directed behavior and reduced capacity to regulate negative emotions effectively, which are key features of depression. Notably, given the frequently reported *hypo*-connectivity within the FPN-left in mood disorders (Kaiser et al., 2015; Gray et al., 2020), our findings suggest that hyper-connectivity in this network plays a role in the therapeutical effects of ECT, potentially serving as a treatment biomarker. Additionally, the association between *hyper*-connectivity within the FPN-right and depressive symptoms in ECT patients suggests these connectivity abnormalities may serve as state-related markers of depressive symptomatology in mood disorders rather than being attributed to the effects of ECT.

Our findings revealed an altered communication between control system networks, characterized by a *hypo*-connectivity between ECN and FPN and a *hyper*-connectivity between DMN and FPN-right, suggesting a reduced efficiency in network interaction for goal-directed tasks. These findings partially align with previous studies reporting *hyper*-connectivity between DMN and FPN-right following ECT treatment (Porta-Casteràs et al., 2021; Wang et al., 2018). Previous studies have shown that effective cognitive engagement requires the deactivation of DMN hyperactivity and increased activation in control system networks (Anticevic et al., 2010). Thus, failure to downregulate DMN hyperactivity and its interaction with ECN and FPN during cognitive engagement may hinder adaptive disengagement from task-irrelevant processing. This inefficiency could contribute to deficits in cognitive and affective functioning, potentially underlying the clinical and cognitive symptoms of ECT-treated patients (Zarp Petersen et al., 2022; Weissman et al., 2006; Petersen et al., 2024).

Consistent with the literature (Semkovska and McLoughlin, 2010), patients presented a decline in verbal learning and memory from pre- to post-ECT, alongside an improvement in depressive symptoms. However, we found no significant association between the observed rs-FC differences and either cognitive decline or depressive symptom improvement. One possible explanation for the lack of association with verbal learning and memory decline is that ECT-related cognitive side-effects may involve distinct neural circuits. For instance, previous studies found that memory impairments were significantly associated with disruptions in connectivity between cortical networks, such as DMN and FPN, and subcortical regions, like the hippocampus and thalamus (Wei et al., 2018; Wang et al., 2020; Qi et al., 2022). In contrast, the identified cluster within the FPN-left was located in the left middle occipital gyrus, a region not typically associated with verbal memory processing. Instead, altered activity in the left dlPFC has been commonly associated with working memory impairments in mood disorders (Zarp Petersen et al., 2022; Miskowiak and Petersen, 2019). Regarding the lack of association with depressive symptoms improvement, our findings align with previous research showing that ECT-induced rs-FC changes do not consistently correlate with clinical outcomes (Wei et al., 2018; Wolf et al., 2016; Thomann et al., 2017). This finding suggests that the neuromodulatory effects of ECT may not always translate to specific cognitive or mood improvements. Further longitudinal studies with comprehensive assessments of depressive symptoms, cognition, and brain function in these networks before and post-ECT are needed to explore this connection in greater depth.

The findings have several implications. Firstly, the study provides novel insights into the differences in resting state-based brain connectivity patterns and their associations with depressive symptoms severity between mood disorder patients with treatment-resistant depression after eight sessions of ECT and patients in partial or full remission with standard pharmacological treatment. Notably, these insights are drawn from a younger sample without severe psychosis or acute suicidal risk, which may limit the generability of the findings. The observed *hyper*-connectivity within right FPN and DMN appears to be mainly driven by mood symptom severity rather than reflecting a marker of ECT treatment per se. Of particular importance, differences in rs-FC within the FPN-left and between ECN and FPN appear independent of depressive symptoms, suggesting they may serve as potential ECT-related markers. However, they did not correlate with the observed verbal learning and memory side effects in ECT-treated patients. Therefore, further research, including MRI pre-ECT data, is necessary to conclusively establish this lack of association between cognitive side effects and functional changes in those networks. Despite this, our results suggest that add-on pro-cognitive treatments targeting these FPN rs-FC abnormalities may prove helpful in alleviating the adverse cognitive effects of ECT. Future neuroimaging research is encouraged to explore longitudinal FC effects of ECT to identify biomarkers that predict – and are related to - treatment response and the likelihood of experiencing cognitive side effects in patients undergoing ECT.

A key limitation was the cross-sectional design employed in the study, along with the absence of longitudinal rs-fMRI data for the ECT-treated group, which impedes the drawing of causal conclusions regarding the neural correlates in patients with mood disorders after ECT, as well as their underlying cognitive impairments. Future studies should collect rs-fMRI data both pre- and post-ECT to identify causal effects of ECT treatment and to better understand the associated cognitive impairments. However, we had clinical and neuropsychological data at both pre-and post-ECT, which allowed us to compute the cognitive change scores for several cognitive domains such as verbal learning and memory performance. Furthermore, the study sample was pooled from three pre-existing studies with different aims, and the findings should be considered exploratory. In future research, new clinical, cognitive, and rs-fMRI data should be collected from a single cohort using a standardized protocol to ensure a more consistent and reliable dataset. Ethical considerations prevented us from including ECT patients with severe psychotic depression who were unable to provide informed consent or those at acute suicidal risk. While ethically necessary, this exclusion may limit the generalizability of our findings, particularly for patients with more severe clinical presentations. Additionally, our sample consisted of relatively younger populations compared to other studies investigating ECT-induced rs-FC changes, which may further restrict the applicability of our results to older clinical populations. Future studies should include patients across a broader age range to enhance the applicability of the results to the general population. Although none of the patients exhibited psychotic symptoms at the time of the scan, it was a limitation that we lacked data on their history of psychotic symptoms. Such information could have influenced the observed brain functional differences between ECT and Non-ECT groups (Brandt et al., 2014). Additionally, we did not have data on anxiety symptom severity or the presence of co-occurring mental disorders within the patient sample, both of which could potentially have influenced the results. Future studies should incorporate comprehensive assessments of psychotic and anxiety symptoms, as well as systematically evaluate the presence of co-occurring mental disorders. Another limitation that may contribute to the difference between our and prior findings is that all ECT patients were assessed after eight sessions of ECT. This differs from other ECT studies, where patients are typically assessed after undergoing a full course of treatment sessions, usually up to 12 sessions (Porter et al., 2020). However, this consistent timepoint for fMRI and cognition assessments ensured a uniform intervention for all ECT patients in the study, and eight sessions were sufficient to induce observable changes in the brain (Gryglewski et al., 2019). In addition, the use of psychotropic medications may have influenced the network connectivity differences observed between patients (Torrent et al., 2011; Sabater et al., 2016; Dias et al., 2012; Ilzarbe and Vieta, 2023). However, our post hoc exploratory analyses did not reveal a significant effect of medication on the identified network connectivity patterns, suggesting that the network dysfunction detected in ECT patients was not solely attributed to their medication status.

## 5 Conclusions

This exploratory rs-fMRI study shows that ECT patients exhibited increased connectivity within the FPN-left and reduced connectivity between central executive networks, like ECN and FPN, compared to non-ECT patients. These connectivity differences were not associated with depressive symptoms and showed no correlation with decline in verbal learning and memory or improvements in depressive symptoms among ECT patients.

## Funding sources

This work was supported by by a five-year 10.13039/501100003554Lundbeck Foundation Fellowship to KWM (R215-2015-4121).

## Declaration of competing interest

KWM reports receiving consultancy fees from Lundbeck, Gedeon Richter and Angelini in the past four years. LVK has within the past four years been a consultant for Lundbeck and Teva. JZP has within the last three years received honoraria from Lundbeck Pharma A/S. EV has received a grant and served as a consultant, advisor or CME speaker for the following entities: AB-Biotics, Abbott, 10.13039/100007819Allergan, 10.13039/501100006546Angelini, 10.13039/501100002975Dainippon Sumitomo Pharma, Ferrer, 10.13039/501100003358Gedeon Richter, Janssen, 10.13039/501100013327Lundbeck, 10.13039/100019120Otsuka, Sage, Sanofi-Aventis, 10.13039/100009655Sunovion, Takeda, all of them unrelated to the current work. GMK has received honoraria as a speaker for Sage Therapeutics and H. Lundbeck, and as an advisor for Onsero and Sanos. LF, AYN, MBJ, JM, PMF, JR report no conflicts of interest.

## References

[bib1] Anticevic A., Repovs G., Shulman G.L., Barch D.M. (2010). When less is more: TPJ and default network deactivation during encoding predicts working memory performance. Neuroimage.

[bib2] Beckmann C.F., DeLuca M., Devlin J.T., Smith S.M. (2005). Investigations into resting-state connectivity using independent component analysis. Philos. Trans. R. Soc. Lond. B Biol. Sci..

[bib3] Beckmann C., Mackay C., Filippini N., Smith S. (2009). Group comparison of resting-state FMRI data using multi-subject ICA and dual regression. Neuroimage.

[bib4] Belge J.B., Mulders P.C.R., Oort J.V. (2021). Movement, mood and cognition: preliminary insights into the therapeutic effects of electroconvulsive therapy for depression through a resting-state connectivity analysis. J. Affect. Disord..

[bib5] Berman M.G., Peltier S., Nee D.E., Kross E., Deldin P.J., Jonides J. (2011). Depression, rumination and the default network. Soc. Cognit. Affect Neurosci..

[bib6] Borkowski J.G., Benton A.L., Spreen O. (1967). Word fluency and brain damage. Neuropsychologia.

[bib7] Brandl F., Weise B., Mulej Bratec S. (2022). Common and specific large-scale brain changes in major depressive disorder, anxiety disorders, and chronic pain: a transdiagnostic multimodal meta-analysis of structural and functional MRI studies. Neuropsychopharmacol.

[bib8] Brandt C.L., Eichele T., Melle I., Sundet K., Server A., Agartz I. (2014). Working memory networks and activation patterns in schizophrenia and bipolar disorder: comparison with healthy controls. Br. J. Psychiatry.

[bib9] Connolly C.G., Wu J., Ho T.C. (2013). Resting-state functional connectivity of subgenual anterior cingulate cortex in depressed adolescents. Biol. Psychiatry.

[bib10] Dias V.V., Balanzá-Martinez V., Soeiro-de-Souza M.G., Moreno R.A., Figueira M.L., Machado-Vieira R. (2012). Pharmacological approaches in bipolar disorders and the impact on cognition: a critical overview: bipolar disorder treatment and cognition. Acta Psychiatr. Scand..

[bib11] Fortea L., Ysbaek-Nielsen A.T., Macoveanu J. (2023). Aberrant resting-state functional connectivity underlies cognitive and functional impairments in remitted patients with bipolar disorder. Acta Psychiatr. Scand..

[bib12] Fox P.T., Lancaster J.L. (2002). Mapping context and content: the BrainMap model. Nat. Rev. Neurosci..

[bib13] Fujita A., Nakaaki S., Segawa K. (2006). Memory, attention, and executive functions before and after sine and pulse wave electroconvulsive therapies for treatment-resistant major depression. J. ECT.

[bib14] Gray J.P., Müller V.I., Eickhoff S.B., Fox P.T. (2020). Multimodal abnormalities of brain structure and function in major depressive disorder: a meta-analysis of neuroimaging studies. Am. J. Psychiatr..

[bib15] Griffanti L., Salimi-Khorshidi G., Beckmann C.F., Auerbach J., Douaud G., Sexton C.E. (2014). ICA-based artefact and accelerated fMRI acquisition for improved Resting State Network imaging. Neuroimage.

[bib16] Gryglewski G., Baldinger-Melich P., Seiger R., Godbersen G.M., Michenthaler P., Klöbl M. (2019). Structural changes in amygdala nuclei, hippocampal subfields and cortical thickness following electroconvulsive therapy in treatment-resistant depression: longitudinal analysis. Br. J. Psychiatry.

[bib17] Hamilton M. (1960). A rating scale for depression. J. Neurol. Neurosurg. Psychiatry.

[bib18] Hammershøj L.G., Petersen J.Z., Jensen H.M., Jørgensen M.B., Miskowiak K.W. (2022). Cognitive adverse effects of electroconvulsive therapy: a discrepancy between subjective and objective measures?. J. ECT.

[bib19] Husain S.S., Kevan I.M., Linnell R., Scott A.I.F. (2004). Electroconvulsive therapy in depressive illness that has not responded to drug treatment. J. Affect. Disord..

[bib20] Ilzarbe L., Vieta E. (2023). The elephant in the room: medication as confounder. Eur. Neuropsychopharmacol..

[bib21] Jenkinson M., Beckmann C.F., Behrens T.E.J., Woolrich M.W., Smith S.M. (2012). Fsl. Neuroimage.

[bib22] Kaiser R.H., Andrews-Hanna J.R., Wager T.D., Pizzagalli D.A. (2015). Large-Scale network dysfunction in major depressive disorder: a meta-analysis of resting-state functional connectivity. JAMA Psychiatry.

[bib23] Kellner C.H., Greenberg R.M., Murrough J.W., Bryson E.O., Briggs M.C., Pasculli R.M. (2012). ECT in treatment-resistant depression. Aust. J. Pharm..

[bib24] Kessing L.V., Munkholm K., Faurholt-Jepsen M., Miskowiak K.W., Nielsen L.B., Frikke-Schmidt R. (2017). The Bipolar Illness Onset study: research protocol for the BIO cohort study. BMJ Open.

[bib25] Landry M., Moreno A., Patry S., Potvin S., Lemasson M. (2021). Current practices of electroconvulsive therapy in mental disorders: a systematic review and meta-analysis of short and long-term cognitive effects. J. ECT.

[bib26] Lecrubier Y., Sheehan D.V., Weiller E., Amorim P., Bonora I., Sheehan K.H. (1997). The Mini International Neuropsychiatric Interview (MINI): a short diagnostic structured interview: reliability and validity according to the CIDI. Eur. Psychiatry.

[bib27] Macoveanu J., Craciun S., Ketterer-Sykes E.B., Ysbæk-Nielsen A.T., Zarp J., Kessing L.V. (2024). Amygdala and hippocampal substructure volumes and their association with improvement in mood symptoms in patients with mood disorders undergoing electroconvulsive therapy. Psychiatry Res. Neuroimaging..

[bib28] Mervaala E., Könönen M., Föhr J. (2001). SPECT and neuropsychological performance in severe depression treated with ECT. J. Affect. Disord..

[bib29] Miskowiak K.W., Petersen C.S. (2019). Neuronal underpinnings of cognitive impairment and - improvement in mood disorders. CNS Spectr..

[bib30] Miskowiak K.W., Petersen J.Z., Macoveanu J., Ysbæk-Nielsen A.T., Lindegaard I.A., Cramer K. (2024). Effect of erythropoietin on cognitive side-effects of electroconvulsive therapy in depression: a randomized, double-blind, placebo-controlled trial. Eur. Neuropsychopharmacol..

[bib31] Mulders P.C.R., van Eijndhoven P.F.P., Pluijmen J., Schene A.H., Tendolkar I., Beckmann C.F. (2016). Default mode network coherence in treatment-resistant major depressive disorder during electroconvulsive therapy. J. Affect. Disord..

[bib32] Nelson H.E., O'Connell A. (1978). Dementia: the estimation of premorbid intelligence levels using the new Adult reading test. Cortex.

[bib33] Office AG (1944).

[bib34] Ott C.V., Vinberg M., Bowie C.R. (2018). Effect of action-based cognitive remediation on cognition and neural activity in bipolar disorder: study protocol for a randomized controlled trial. Trials.

[bib35] Perera T.D., Luber B., Nobler M.S., Prudic J., Anderson C., Sackeim H.A. (2004). Seizure expression during electroconvulsive therapy: relationships with clinical outcome and cognitive side effects. Neuropsychopharmacol.

[bib36] Perrin J.S., Merz S., Bennett D.M. (2012). Electroconvulsive therapy reduces frontal cortical connectivity in severe depressive disorder. Proc. Natl. Acad. Sci. USA..

[bib37] Petersen J.Z., Schmidt L.S., Vinberg M., Jørgensen M.B., Hageman I., Ehrenreich H. (2018). Effects of recombinant human erythropoietin on cognition and neural activity in remitted patients with mood disorders and first-degree relatives of patients with psychiatric disorders: a study protocol for a randomized controlled trial. Trials.

[bib38] Petersen J.Z., Macoveanu J., Ysbæk-Nielsen A.T., Kessing L.V., Jørgensen M.B., Miskowiak K.W. (2024). Neural correlates of episodic memory decline following electroconvulsive therapy: an exploratory functional magnetic resonance imaging study. J. Psychopharmacol..

[bib39] Porta-Casteràs D., Cano M., Camprodon J.A. (2021). A multimetric systematic review of fMRI findings in patients with MDD receiving ECT. Prog. Neuro Psychopharmacol. Biol. Psychiatr..

[bib40] Porter R.J., Baune B.T., Morris G., Hamilton A., Bassett D., Boyce P. (2020). Cognitive side-effects of electroconvulsive therapy: what are they, how to monitor them and what to tell patients. BJPsych Open.

[bib41] Posternak M.A., Young D., Sheeran T., Chelminski I., Franklin C.L., Zimmerman M. (2004). Assessing past treatment history: test-retest reliability of the Treatment Response to Antidepressant Questionnaire. J. Nerv. Ment. Dis..

[bib42] Qi S., Calhoun V.D., Zhang D., Miller J., Deng Z.D., Narr K.L. (2022). Links between electroconvulsive therapy responsive and cognitive impairment multimodal brain networks in late-life major depressive disorder. BMC Med..

[bib43] Randolph C., Tierney M.C., Mohr E., Chase T.N. (1998). The repeatable battery for the assessment of neuropsychological status (RBANS): preliminary clinical validity. J. Clin. Exp. Neuropsychol..

[bib44] Runia N., Yücel D.E., Lok A. (2022). The neurobiology of treatment-resistant depression: a systematic review of neuroimaging studies. Neurosci. Biobehav. Rev..

[bib45] Sabater A., García-Blanco A.C., Verdet H.M., Sierra P., Ribes J., Villar I. (2016). Comparative neurocognitive effects of lithium and anticonvulsants in long-term stable bipolar patients. J. Affect. Disord..

[bib46] Sackeim H.A. (2001). The definition and meaning of treatment-resistant depression. J. Clin. Psychiatry.

[bib47] Sahakian B.J., Owen A.M. (1992). Computerized assessment in neuropsychiatry using CANTAB: discussion paper. J. R. Soc. Med..

[bib48] Salimi-Khorshidi G., Douaud G., Beckmann C.F., Griffanti L., Smith S.M. (2014). Automatic denoising of functional MRI data: combining independent component analysis and hierarchical fusion of classifiers. Neuroimage.

[bib49] Schat A., Van Den Broek W.W., Mulder P.G.H., Birkenhäger T.K., Van Tuijl R., Murre J.M.J. (2007). Changes in everyday and semantic memory function after electroconvulsive therapy for unipolar depression. J. ECT.

[bib50] Schmidt M. (1996).

[bib51] Schmidt L.S., Petersen J.Z., Vinberg M., Hageman I., Olsen N.V., Kessing L.V. (2018). Erythropoietin as an add-on treatment for cognitive side effects of electroconvulsive therapy: a study protocol for a randomized controlled trial. Trials.

[bib52] Semkovska M., McLoughlin D.M. (2010). Objective cognitive performance associated with electroconvulsive therapy for depression: a systematic review and meta-analysis. Biol. Psychiatry.

[bib53] Sha Z., Wager T.D., Mechelli A., He Y. (2019). Common dysfunction of large-scale neurocognitive networks across psychiatric disorders. Biol. Psychiatry.

[bib54] Sienaert P. (2016). Based on a true story? The portrayal of ECT in international movies and television programs. Brain Stimul..

[bib55] Smith S.M., Fox P.T., Miller K.L., Glahn D.C., Fox P.M., Mackay C.E. (2009). Correspondence of the brain's functional architecture during activation and rest. Proc. Natl. Acad. Sci. USA..

[bib56] Smith S.M., Vidaurre D., Beckmann C.F. (2013). Functional connectomics from resting-state fMRI. Trends Cognit. Sci..

[bib57] Snyder H.R. (2013). Major depressive disorder is associated with broad impairments on neuropsychological measures of executive function: a meta-analysis and review. Psychol. Bull..

[bib58] Thomann P.A., Wolf R.C., Nolte H.M., Hirjak D., Hofer S., Seidl U. (2017). Neuromodulation in response to electroconvulsive therapy in schizophrenia and major depression. Brain Stimul..

[bib59] Torrent C., Martinez-Arán A., Daban C., Amann B., Balanzá-Martínez V., del Mar Bonnín C. (2011). Effects of atypical antipsychotics on neurocognition in euthymic bipolar patients. Compr. Psychiatry.

[bib60] UK ECT Review Group (2003). Efficacy and safety of electroconvulsive therapy in depressive disorders: a systematic review and meta-analysis. Lancet.

[bib61] Wang J., Wei Q., Wang L. (2018). Functional reorganization of intra- and internetwork connectivity in major depressive disorder after electroconvulsive therapy. Hum. Brain Mapp..

[bib62] Wang D., Tian Y., Li M., Dahmani L., Wei Q., Bai T. (2020). Functional connectivity underpinnings of electroconvulsive therapy-induced memory impairments in patients with depression. Neuropsychopharmacology.

[bib63] Wechsler D. (1997).

[bib64] Wei Q., Bai T., Chen Y. (2018). The changes of functional connectivity strength in electroconvulsive therapy for depression: a longitudinal study. Front. Neurosci..

[bib65] Weissman D.H., Roberts K.C., Visscher K.M., Woldorff M.G. (2006). The neural bases of momentary lapses in attention. Nat. Neurosci..

[bib67] Winkler A.M., Ridgway G.R., Webster M.A., Smith S.M., Nichols T.E. (2014). Permutation inference for the general linear model. Neuroimage.

[bib68] Wolf R.C., Nolte H.M., Hirjak D., Hofer S., Seidl U., Depping M.S. (2016). Structural network changes in patients with major depression and schizophrenia treated with electroconvulsive therapy. Eur. Neuropsychopharmacol..

[bib69] Young R.C., Biggs J.T., Ziegler V.E., Meyer D.A. (1978). A rating scale for Mania: reliability, validity and sensitivity. Br. J. Psychiatr..

[bib70] Yrondi A., Péran P., Sauvaget A., Schmitt L., Arbus C. (2018). Structural–functional brain changes in depressed patients during and after electroconvulsive therapy. Acta Neuropsychiatr..

[bib71] Zarp Petersen J., Varo C., Skovsen C.F. (2022). Neuronal underpinnings of cognitive impairment in bipolar disorder: a large data-driven functional magnetic resonance imaging study. Bipolar Disord..

