# Supplementary material for: Functional connectivity differences in patients with mood disorders: an exploratory fMRI study comparing electroconvulsive therapy with pharmacological treatment
Source: Neurosci Appl. 2025 Jun 11;4:105522. doi: 10.1016/j.nsa.2025.105522 (PMC12664636; doi:10.1016/j.nsa.2025.105522)
Supplement: Multimedia component 1 [file mmc1.docx]

**Supplementary Materials**

**Methods and Materials**

*MRI acquisition parameters*

We acquired blood-oxygen-level-dependent (BOLD) fMRI during resting-state with a T2*-weighted gradient echo spiral echo-planar (EPI) sequence with the following parameters: echo time (TE) = 30 ms, repetition time (TR) = 2 s, and flip angle = 90°. The acquisition included 300 volumes, each consisting of 32 slices, slice thickness = 3 mm with 25% gaps in-between and a field of view (FOV) = 230 × 230 mm using a 64 × 64 grid. To register the BOLD images to the MNI standard space (see below), T1-weighted structural images were acquired (TR = 1900 ms; TE = 2.58 ms; flip angle = 9°; distance factor = 50%; FOV = 230 × 230 mm; slice thickness = 0.9 mm). Furthermore, a standard B0 field map sequence was acquired with the same FOV and resolution as the fMRI sequence (TR = 400 ms; TE = 7.38 ms; flip angle = 60°) to correct for geometric distortions correction of the BOLD images. We ascertained image quality by visual inspection of all individual participant images.

**Results**

*RSFC analysis*

(i) Effect of ECT without adjusting for depressive symptoms

When exploring the connectivity within DMN, FPN-right, and FPN-left at the voxel-wise level, we found that ECT patients showed greater positive connectivity within the DMN in one big cluster, expanding to the bilateral ACC and PFC, and within both FPN-right and left compared to non-ECT patients (Figure S2, Table S2). The significant cluster of FPN-right was located in the right orbitofrontal cortex (OFC) and dorsolateral prefrontal cortex (dlPFC). The three significant clusters of FPN-left were located in the left OFC and dlPFC, and the supramarginal gyrus extended to the lateral occipital cortex. All the obtained clusters prevailed when adjusting for differences in medication status between patient groups.

Between-RSN connectivity analyses showed that ECT patients presented higher positive connectivity between DMN and FPN-right (*p*<0.024), lower positive connectivity between ECN and FPN-left and right (*p*<0.005), and lower negative connectivity between ECN and DMN (r=-2.2 vs. -0.6, *p*<0.019) compared to non-ECT patients. Only the lower connectivity between ECN and FPN-left survived FWER correction. When adjusting for anti-depressive and antipsychotic medication, the hypoconnectivity between ECN and FPN-left, FPN-right, and DMN prevailed significantly, but not the hyperconnectivity between DMN and FPN-right.

**Figure S1.** Selected component from ICA analysis with all subjects representing the four resting state networks of interest (DMN, ECN, FPN-left, and FPN-right) at Z > 3.


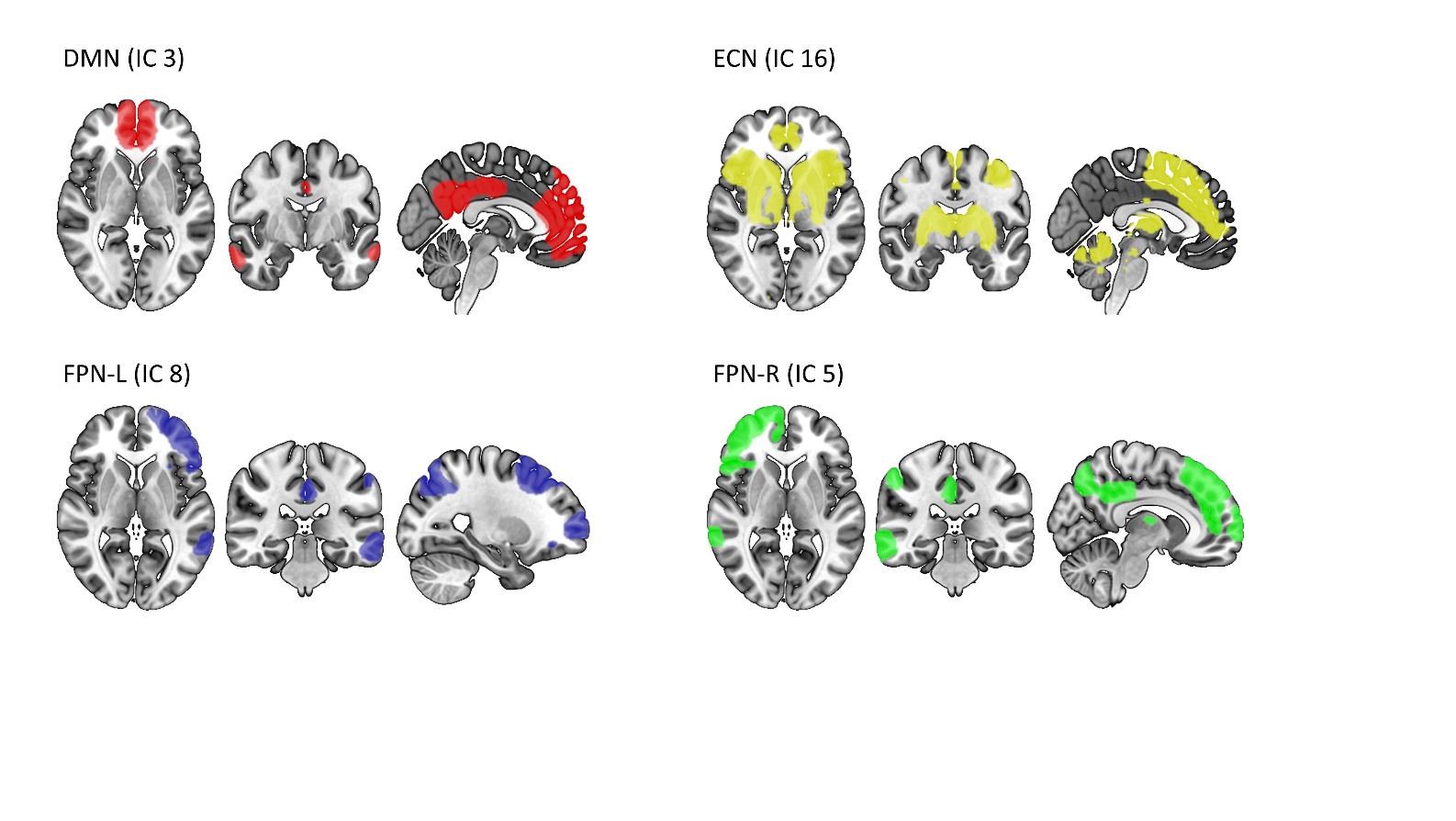


Footnote: DMN: Default mode network; ECN: executive central network; FPN: frontoparietal network; ICA: independent component analysis; L: left; R: right.

**Figure S2.** Analysis of within-network connectivity between ECT and no-ECT patients for DMN, FPN-right, and FPN-left, without adjusting for mood symptoms. A) Brain regions showing significantly higher functional connectivity within the studied network, B) Boxplot comparing integrity values between groups.
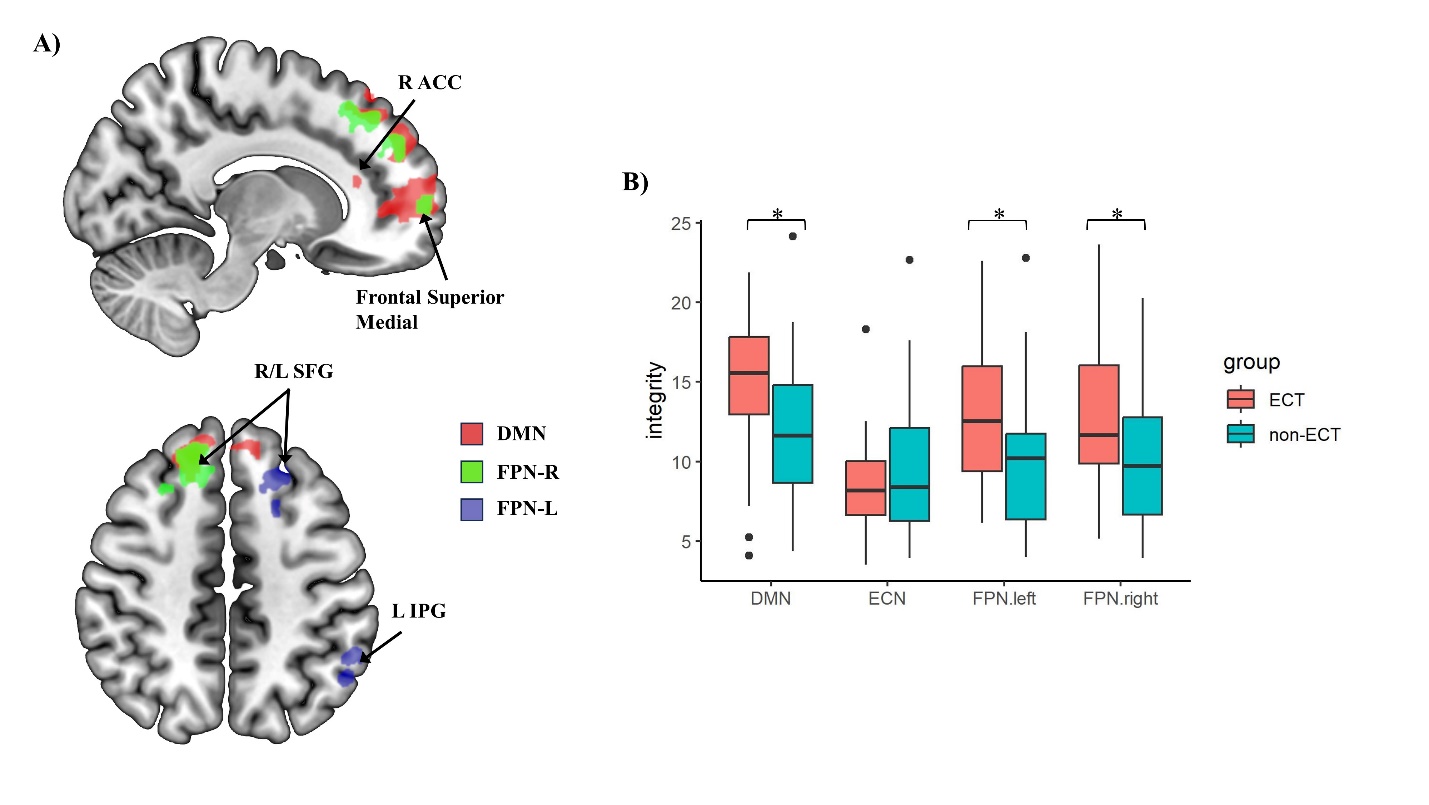


Footnote: DMN: Default mode network; ECN: executive central network; ECT: electroconvulsive therapy; FPN: frontoparietal network; L: left; R: right.

**Supplementary Table S1.** Differences in depressive symptoms and cognitive performance in the ECT patient group pre-and post-treatment. Cognitive measures are presented as Z-scores.

|  | Pre-ECT | Post-ECT | change | t | p |
| --- | --- | --- | --- | --- | --- |
| HDRS-17  *mean (SD)* | 27.29 (5.6) | 17.81 (9.3) | 9.62 (9.5) | 5.05 | <0.001* |
| Processing speed | -1.29 | -1.34 | -0.05 | -0.269 | 0.395 |
| Verbal Learning | -0.82 | -1.16 | -0.35 | 1.89 | 0.034* |
| Working memory | -1.15 | -1.16 | -0.01 | 0.067 | 0.473 |
| Attention | -0.72 | -0.39 | 0.31 | 1.57 | 0.930 |
| Global | -0.99 | -1.04 | -0.04 | 0.32 | 0.374 |

* Statistically significant, α ≤ 0.05

Footnote: ECT: electroconvulsive therapy; HDRS-17: Hamilton Depression Rating Scale 17-items; SD: standard deviation.

**Table S2.** Regions showing higher resting-state functional connectivity within the studied networks between ECT-treated patients with mood disorders and those with typical pharmacological treatment without adjusting for depressive symptoms. Results were cluster-corrected, and significance was assessed based on the number of studied components (4 components: α ≤ 0.0125).

|  | **Regions** | **t** | **p** | **No. of voxels (cluster size)** | **Peak MNI coordinates** | | |
| --- | --- | --- | --- | --- | --- | --- | --- |
|  |  |  |  |  | **X** | **Y** | **Z** |
| **DMN** |  |  |  |  |  |  |  |
|  | R Frontal Pole / superior frontal gyrus | 5.16 | <0.001 | 2668 | 16 | 64 | 4 |
|  |  |  |  |  |  |  |  |
| **FPN-R** |  |  |  |  |  |  |  |
|  | R frontal pole / superior frontal gyrus | 4.59 | <0.001 | 2008 | 38 | 48 | -8 |
|  |  |  |  |  |  |  |  |
| **FPN-L** |  |  |  |  |  |  |  |
|  | L supramarginal gyrus | 5.19 | 0.002 | 244 | -38 | -48 | 32 |
|  | L superior frontal gyrus | 4.48 | 0.004 | 194 | -20 | 22 | 54 |
|  | L frontal pole | 4.63 | 0.004 | 108 | -36 | 48 | -8 |
|  |  |  |  |  |  |  |  |

Footnote: DMN: Default mode network; ECN: executive central network; ECT: electroconvulsive therapy; FPN: frontoparietal network; L: left; R: right.

**Table S3.** Regions showing higher resting-state functional connectivity within the left frontoparietal network (FPN-L) between ECT-treated patients with mood disorders and those with typical pharmacological treatment adjusting for depressive symptoms and different medications. Results were cluster-corrected, with p < 0.05.

|  | **Regions** | **t** | **p** | **No. of voxels (cluster size)** | **Peak MNI coordinates** | | |
| --- | --- | --- | --- | --- | --- | --- | --- |
| **Medication** |  |  |  |  | **X** | **Y** | **Z** |
| **Anticonvulsants** |  |  |  |  |  |  |  |
|  | Left middle occipital gyrus | 3.67 | 0.034 | 52 | -38 | -70 | 26 |
|  |  |  |  |  |  |  |  |
| **Antidepressants** |  |  |  |  |  |  |  |
|  | Left middle occipital gyrus | 2.78 | 0.039 | 53 | -38 | -70 | 26 |
|  |  |  |  |  |  |  |  |
| **Antipsychotics** |  |  |  |  |  |  |  |
|  | Left middle occipital gyrus | 3.74 | 0.028 | 66 | -38 | -70 | 30 |
|  |  |  |  |  |  |  |  |
| **Benzodiazepines** |  |  |  |  |  |  |  |
|  | Left middle occipital gyrus | 3.94 | 0.024 | 114 | -38 | -70 | 26 |
|  |  |  |  |  |  |  |  |
| **Lithium** |  |  |  |  |  |  |  |
|  | Left middle occipital gyrus | 4.38 | 0.023 | 106 | -38 | -70 | 26 |
|  |  |  |  |  |  |  |  |

Footnote: DMN: Default mode network; ECN: executive central network; ECT: electroconvulsive therapy; FPN: frontoparietal network; L: left; R: right.

**Table S4.** Comparison of between-network connectivity between non-ECT treated patients (n=36) and those patients from the ECT-treated group who responded to ECT (n=12), adjusted for depressive symptoms. Significance was set at uncorrected p ≤ 0.05.

| **Pair of RSN** | **t** | **b** | **p** |
| --- | --- | --- | --- |
|  |  |  |  |
| DMN - ECN | -2.1 | -2.26 | 0.04 |
| FPN-R - ECN | -2.64 | -2.87 | 0.01 |
| FPN-L - ECN | -3.29 | -4 | 0.002 |
|  |  |  |  |

Footnote: DMN: Default mode network; ECN: executive central network; ECT: electroconvulsive therapy; FPN: frontoparietal network; L: left; R: right; RSN: resting-state network.
